# Aggravation from Noise

**Published:** 1887-12

**Authors:** S. L. G. Leggett


### AGGRAVATION FROM NOISE.

EDITORS OF THE HOMŒOPATHIC PHYSICIAN:—If Dr. Durand, who inquires in the October number (page 389) for a remedy for backache, will turn to Bœnninghausen's *Repertory* he will find the following:

Remedies having dull pain in sacrum: AGAR., Alum., ANT. C., Bar., Bov., *Calc.*, Canth., Carb. v., Caust., Chin., Cic., Dros., Hep., IGNAT., LAUR., Led., Merc., Phos., *Ph. ac.*, *Plat.*, Plumb., Rhus, Sil., Spig., *Thuj.*, Veratr., Vit., Zinc.

Turn to *agg.* from noise of the above remedies.

Alum, Bor., CALC., Caust., Chin., Ignat., Merc., PH. AC., PLAT., Sil., SPIG., Zinc.

In Lee's *Repertory of Characteristics* sound is divided into several heads, and one must take such heading as seems analogous to the "shrill" sound mentioned, as each sound is represented by a tone or its variation in the musical scale. CALC. should cover the case.

Lee's *Repertory* under *agg.* from noise: Calc., Ignat., Ph., ac., Plat., Spig.

Music—Calc., Ph. ac.

Violin—Calc.

Piano—Calc., Zinc. S. L. G. LEGGETT.

